# Extracellular 5′-methylthioadenosine inhibits intracellular symmetric dimethylarginine protein methylation of FUSE-binding proteins

**DOI:** 10.1016/j.jbc.2022.102367

**Published:** 2022-08-11

**Authors:** Baiqing Tang, Hyung-Ok Lee, Sapna Gupta, Liqun Wang, Alison M. Kurimchak, James S. Duncan, Warren D. Kruger

**Affiliations:** 1 Molecular Therapeutics Program, Fox Chase Cancer Center, Philadelphia, Pennsylvania, USA; 2 Cancer Signaling and Epigenetics Program, Fox Chase Cancer Center, Philadelphia, Pennsylvania, USA

**Keywords:** protein methylation, metabolism, methionine, DNA-binding protein, proteomics, aDMA, asymmetric dimethylarginine, CE, collision energy, Dox, doxycycline, FUSE, far upstream binding sequence element, HTM+, HT1080 cells expressing MTAP, HTM−, HT1080 cells lacking MTAP, IP, immunoprecipitation, MMA, monomethylarginine, MTA, 5′-deoxy-5′-methylthioadenosine, MTAP, methylthioadenosine phosphorylase, PRMT, protein arginine methyltransferase, RIPA, radioimmunoprecipitation assay, sDMA, symmetric dimethylarginine

## Abstract

Methylthioadenosine phosphorylase (MTAP) is a key enzyme in the methionine salvage pathway that converts the polyamine synthesis byproduct 5′-deoxy-5′-methylthioadenosine (MTA) into methionine. Inactivation of MTAP, often by homozygous deletion, is found in both solid and hematologic malignancies and is one of the most frequently observed genetic alterations in human cancer. Previous work established that MTAP-deleted cells accumulate MTA and contain decreased amounts of proteins with symmetric dimethylarginine (sDMA). These findings led to the hypothesis that accumulation of intracellular MTA inhibits the protein arginine methylase (PRMT5) responsible for bulk protein sDMAylation. Here, we confirm that MTAP-deleted cells have increased MTA accumulation and reduced protein sDMAylation. However, we also show that addition of extracellular MTA can cause a dramatic reduction of the steady-state levels of sDMA-containing proteins in MTAP*+* cells, even though no sustained increase in intracellular MTA is found because of catabolism of MTA by MTAP. We determined that inhibition of protein sDMAylation by MTA occurs within 48 h, is reversible, and is specific. In addition, we have identified two enhancer-binding proteins, FUBP1 and FUBP3, that are differentially sDMAylated in response to MTAP and MTA. These proteins work *via* the far upstream element site located upstream of Myc and other promoters. Using a transcription reporter construct containing the far upstream element site, we demonstrate that MTA addition can reduce transcription, suggesting that the reduction in FUBP1 and FUBP3 sDMAylation has functional consequences. Overall, our findings show that extracellular MTA can inhibit protein sDMAylation and that this inhibition can affect FUBP function.

Methylthioadenosine phosphorylase (MTAP) is a key enzyme in the methionine salvage pathway that converts the polyamine byproduct 5′-dideoxy-5′-methylthioadenosine (MTA) into adenine and methylthioribose 1P (Fig. S1*A*). It is highly conserved in eukaryotic evolution and expressed in all mammalian tissues at relatively high levels. Forty-three years ago, Toohey (1) first recognized that certain murine leukemia cell lines lacked MTAP activity. Today, we know that loss of MTAP expression is frequent (>10%) in a large number of different human cancers, including glioblastoma, mesothelioma, bladder cancer, pancreatic adenocarcinoma, head and neck cancer, non–small cell lung cancer, melanoma, and diffuse large-cell lymphoma (Fig. S1*B*). Loss of expression is generally because of homozygous deletion of the human chromosome 9p21 region, where MTAP resides near the *CDKN2A/ARF* gene (2, 3). In culture, MTAP-deleted tumor cells excrete large amounts of MTA into the media and have increased intracellular MTA concentrations (4, 5, 6). Functional studies indicate that MTAP functions as a tumor suppressor gene affecting processes involved in soft-agar colony formation, cell migration, invasion, and epithelial-to-mesenchyme transition (6, 7, 8, 9, 10, 11).

The high frequency of MTAP deletion in tumors, along with its ubiquitous expression in normal tissues, has made the MTAP-pathway alteration a potential target of interest for cancer therapy. Three different groups have performed synthetic lethal screens using shRNA to identify genes that have synthetic slow-growth interactions with MTAP deletion (12, 13, 14). All three groups found that MTAP-deleted cells were more sensitive to shRNA knockdown of protein arginine methyltransferase (*PRMT5*) than MTAP*+* cells. *PRMT5* encodes an arginine methyltransferase responsible for the modification of protein arginine residues to symmetric dimethylarginine (sDMA). This post-translational modification tends to occur on arginine residues that are flanked by glycines (GRG), which is different than the RGG motif favored by the asymmetric dimethylarginine (aDMA) and monomethylarginine (MMA) modifications (15). Many sDMA-containing proteins (sDMAylated) are involved in RNA-dependent processes, such as pre-mRNA splicing, polyadenylation, and transcription (16). PRMT5 utilizes S-adenosylmethionine (AdoMet) as a substrate, and MTA acts as a competitive inhibitor of its enzymatic activity (17). It is important to note that complete loss of *PRMT5* is lethal at the cellular level (18). Utilizing antibodies that recognize the sDMA modification, the same three groups also demonstrated that MTAP deletion and *PRMT5* knockdown caused a reduction in overall cellular protein sDMAylation. Other genes identified in these screens include the *PRMT5* interactors *WDR77* and *RIOK1*, along with the *MAT2A*, which encodes S-adenosylmethionine synthetase. The hypothesis put forward to explain these observations is there is some minimal threshold level of protein sDMAylation required for cell viability and that the combination of elevated intracellular MTA, reduced PRMT5 protein level, and/or reduced AdoMet concentrations is sufficient to go below this threshold (Fig. S1*C*).

Here, we describe our laboratory efforts to more fully understand the relationships between MTAP, *PRMT5*, MTA, and protein sDMAylation. Our data show that exposure of MTAP+ cells to extracellular MTA causes decreased protein sDMAylation in the absence of sustained elevated intracellular MTA accumulation and that MTAP+ cells can restore sDMAylation in MTAP*-*deleted cells in *trans*. In addition, we have identified two enhancer-binding proteins, FUBP1 and FUBP3, that show reduced sDMAylation in response to extracellular MTA. Our data suggest that MTA can affect protein sDMAylation by both extracellular and intracellular mechanisms and that these effects can alter cellular physiology.

## Results

### MTAP deletion causes a reduction in protein sDMAylation

**Figure 1:**
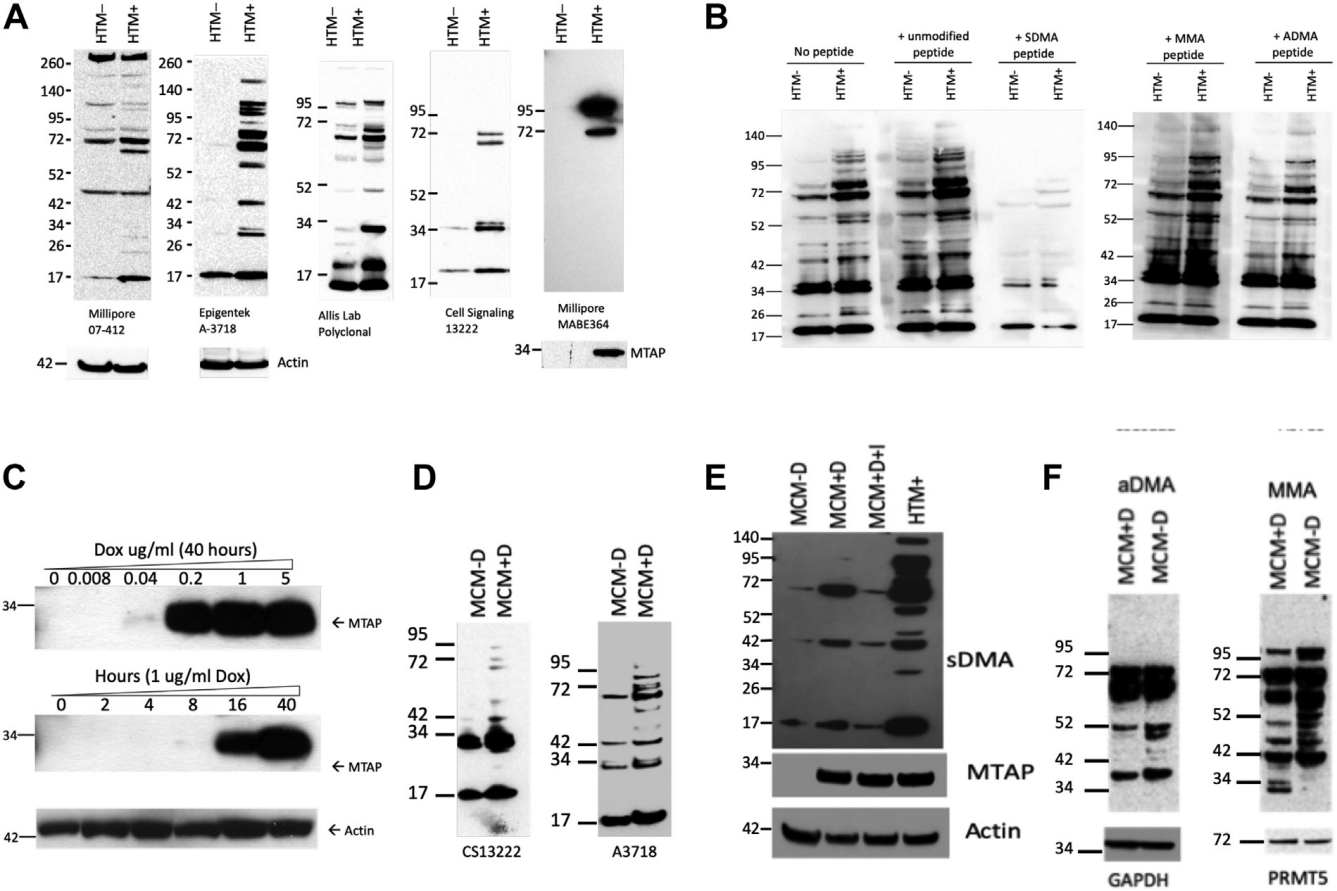
**sDMA decreased in MTAP− cells.***A*, isogenic HTM+ and HTM− cells probed with sDMA-recognizing antibodies from various commercial and academic sources (Fig. S1 for additional information on each antiserum). *B*, Cell-Signaling sDMA antibodies (catalog no.: 13222) probed against HTM+ and HTM− cell lysates in the presence or the absence of indicated competitor peptides. Fivefold molar excess of competitor peptide was added. *C*, Western blot of MCM cell lysates from cells exposed to doxycycline (Dox) at various times or concentrations probed with either α-MTAP or α-actin. *D*, extracts from MCM cells grown in either the presence (+D) or the absence (−D) of Dox probed with two different sDMA-recognizing antiserum. *E*, first two lanes same as *D*, third lane contains induced MCM cells treated with MTAP-specific inhibitor MT-DADMe-ImmA for 48 h. Lane 4 has HTM+ cell extract as positive control. *F*, MCM cells with/without Dox probed with antibodies recognizing either aDMA or MMA. Loading controls are shown below. aDMA, asymmetric dimethylarginine; MTAP, methylthioadenosine phosphorylase; sDMA, symmetric dimethylarginine.

In our initial examination of MTAP status and protein sDMAylation, we utilized a pair of isogenic MTAP+ and MTAP-deleted HT1080 fibrosarcoma cell lines (referred to HTM+ and HTM−) that our laboratory has previously generated (6). These two lines were used in immunoblots that were probed with five different antiserums generated against various peptides containing sDMA (Fig. 1*A*). Each of the antiserums recognized several different bands that were either entirely absent or significantly reduced in intensity in the HTM− relative to the HTM+ cell line. A significant issue that we encountered was that for at least two of the commercial antibodies (Millipore; catalog no.: 07-412 and Epigentek; catalog no.: A-3718), there was significant variability between different lots of the antiserum (Fig. S2). We suspect this might be due to the fact that these are polyclonal serums raised against small peptide antigens containing sDMA and that each “lot” is serum from an individual rabbit.

To confirm that the antiserums were specific for the recognition of sDMA, we performed peptide competition studies, in which a 10 amino acid peptide with an arginine at position 3 was either unmodified or modified with sDMA, aDMA, or MMA. The sequence of this peptide was derived from histone H4 and has been used as an antigen to create sDMA-recognizing antibodies (19). We then added this peptide in a fivefold molar excess to a primary antibody-binding solution containing the CS13222 antiserum and performed Western analysis on HTM+ and HTM− cell lysates. We focused on this antiserum as it is a mixture of four different monoclonal antibodies that recognize sDMA and had the least amount of lot-to-lot variability. Only the sDMA-containing peptide successfully competed for binding, significantly reducing signal for all the bands (Fig. 1*B*). These results confirm that the CS13222 antibody specifically recognizes the sDMA modification and gave us confidence that the bands we were detecting actually contained sDMA.

We next examined the effect of MTAP on protein sDMAylation in an MCF-7 breast adenocarcinoma-derived cell line in which MTAP under control of a doxycycline (Dox)-induced promoter (Fig. 1*C*). As expected, treatment of this cell line with Dox resulted in significantly increased sDMAylation compared with untreated cells (Fig. 1*D*). This finding was further confirmed by showing that sDMAylation caused by Dox was reversed by treatment with the specific MTAP inhibitor, MT-DADMe-ImmA (Fig. 1*E*). We also measured the effect of MTAP on aDMA and MMA in these cells (Fig. 1*F*). We only observed a slight difference in aDMA crossreactivity in response to MTAP status but found that MTAP expression seemed to affect several MMA-specific bands, with some proteins increasing and some decreasing in intensity.

Since both HT1080 and MCF-7 cells are derived from MTAP-deleted cancer cells, we were curious as to what the effect of MTAP loss might be on a nontransformed MTAP*+* cell line. Therefore, we generated clones of mouse 3T3 cells in which MTAP was inactivated using CRISPR technology. Two separate clones both show decreased sDMAylation compared with clones that had intact MTAP (Fig. S3*A*). These results that inactivation of MTAP can reduce protein sDMAylation even in noncancer cell lines.

**Figure 2:**
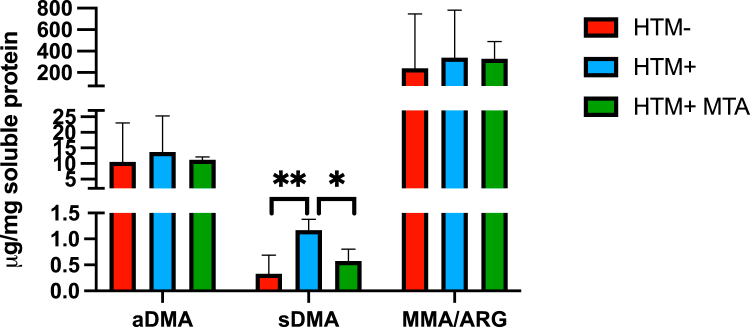
**Assessment of methylated arginine in hydrolyzed protein lysates.** Lysates from HTM−, HTM+, and HTM+ cells treated with 100 mM MTA (48 h) were prepared and then hydrolyzed by XXXYYY. Resulting material was then analyzed using an amino acid analyzer for aDMA, sDMA, and MMA. Note that we were unable to resolve the arginine from the MMA peak, so they are quantitated together. aDMA, asymmetric dimethylarginine; MTA, 5′-deoxy-5′-methylthioadenosine; sDMA, symmetric dimethylarginine.

To quantify how MTAP expression affects overall cellular sDMA content, we measured the amount of methylated arginine in hydrolyzed protein lysates from HTM+ and HTM− cells using an amino acid analyzer (Fig. 2). This technology allowed accurate quantification of sDMA and aDMA, but MMA could not be distinguished from arginine. Overall, there was approximately 30-fold more aDMA than sDMA in cell lysates, indicating that protein aDMAylation is a much more common modification than sDMAylation in HT1080 cells. Furthermore, HTM cells had 62% less sDMA than HTM+ cells, but we did not observe any difference in aDMA or MMA/arginine levels. Treatment of HTM+ cells with extracellular MTA caused a reduction in sDMA but not aDMA or MMA/arginine. These findings show that loss of MTAP or treatment with MTA causes decreased protein sDMAylation.

Finally, we performed cell fractionation studies on HTM− and HTM+ cells to compare the effect of MTAP on protein sDMAylation in cytoplasmic *versus* nuclear proteins (Fig. S3*B*). Both cytoplasmic and nuclear proteins showed reduced levels of sDMAylation in HTM cells.

### PRMT5, but not MAT2A, inhibition causes decreased protein sDMAylation

Since the relationship between MTAP and sDMA was first identified by a synthetic interaction with *PRMT5*, we decided to examine this interaction in our isogenic HTM+ and HTM− cells. Knockdown of *PRMT5* using shRNA confirmed that HTM− cells were somewhat more sensitive than HTM+ cells in a colony formation assay (Fig. S4*A*). We also showed that knockdown of PRMT5 caused decreased protein sDMAylation both in HTM+ and HTM− cells (Fig. S4*B*). This was further confirmed by the use of a specific inhibitor of PRMT5, EPZ015666 (Fig. S4*C*). However, like Mavrakis *et al.* (14), we did not observe any difference in growth inhibition of MTAP+ and MTAP− cells in response to the PRMT5 small-molecule inhibitor EPZ015666 (Fig. S4*D*), suggesting that reduction of PRMT5 protein and inhibition of PRMT5 enzyme activity do not produce identical effects.

We also examined the effect of the MAT2A inhibitor PF9366 in HTM+ and HTM− cells as MAT2A was identified by shRNA screens as having a synthetic growth phenotype by two of the three groups (13, 14). Unlike the PRMT5 inhibitor, we failed to observe any effect of the drug on sDMA levels (Fig. S5*A*). To confirm the drug was working, we measured AdoMet concentrations in treated cell extracts and found they were 79 to 90% lower than in untreated cells (Fig. S5*B*). We also observed no difference in growth inhibition between HTM+ and HTM− cells (Fig. S5*C*). Our findings show that in HT1080 cells, lowering AdoMet levels does not affect by the MTAP genotype.

### External MTA causes decreased protein sDMAylation

**Figure 3:**
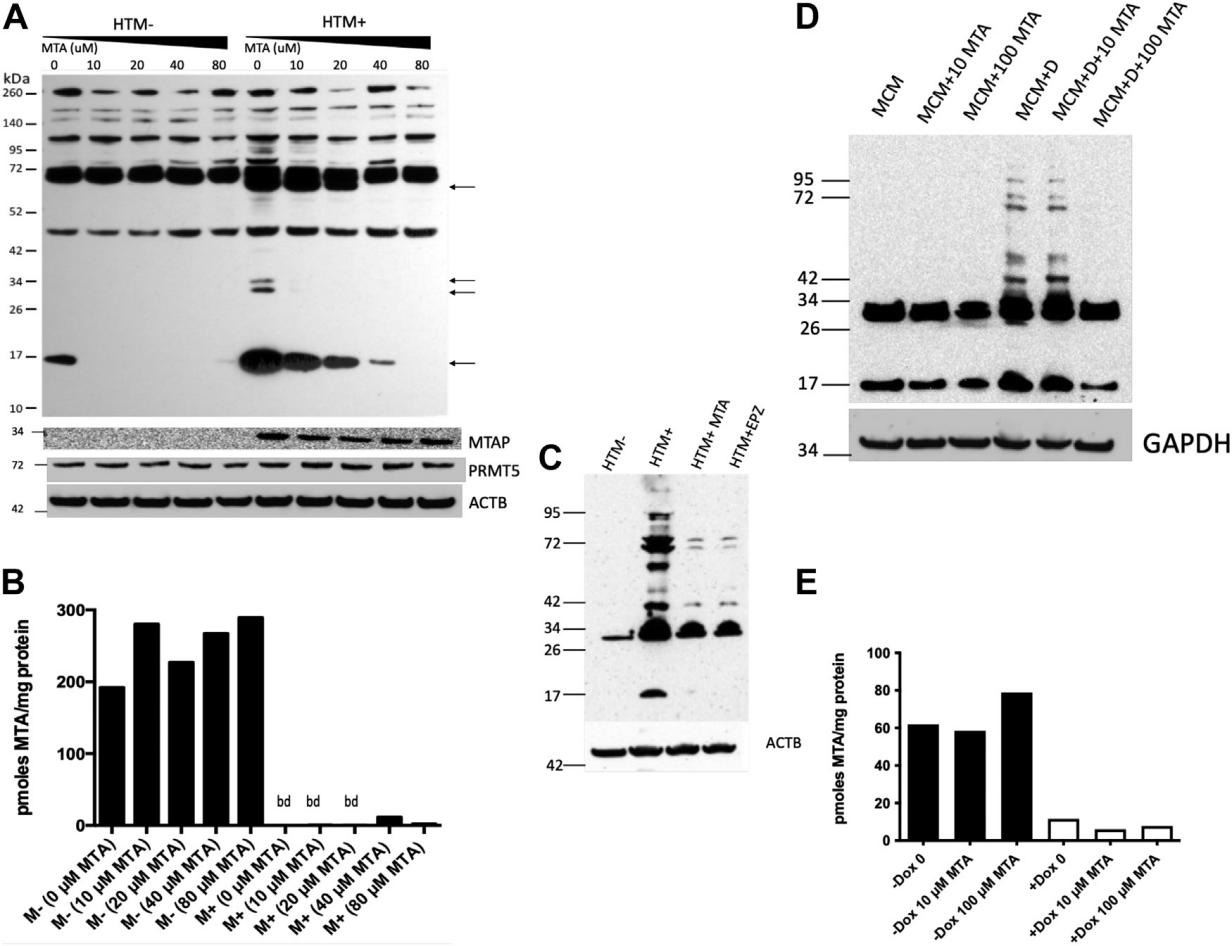
**Inhibition of sDMAylation by extracellular MTA.***A*, cells of indicated genotype were treated for 48 h with indicated amounts of MTA. Extracts were probed with Millipore 07-412 (sDMA, Sym10). *B*, MTA concentrations in extracts from same samples. bd, below detection. *C*, Western blot of HTM− and HTM+ cells treated with either 80 μM MTA or 1.5 μM EPZ015666 for 48 h probed with CS13222 anti-sDMA. *D*, Western blot of MCM cells with and without 1 μg/ml doxycycline (+Dox) and/or indicated amount of MTA probed with CS13222 anti-sDMA. *E*, MTA concentrations in same extracts. MTA, 5′-deoxy-5′-methylthioadenosine; sDMA, symmetric dimethylarginine.

As shown in Figure 2, treatment of HTM+ cells with 80 μM extracellular MTA for 72 h results in decreased protein sDMAylation. To explore this in more depth, we performed a titration experiment in which HTM− and HTM+ cells were treated with varying concentrations of extracellular MTA. We observed a dose-dependent decrease in several proteins that were recognized by the Millipore 07-412 antibody, which was raised against an artificial peptide containing four sDMA repeats (Fig. 3*A*). A similar dose response was also observed using the Epigentek A-3718 antibody (Fig. S6). Surprisingly, the intracellular MTA levels, which are quite low in HTM+ cells, did not increase significantly in response to external MTA despite having reduced sDMA (Fig. 3*B*). Addition of 80 μM MTA to HTM+ cells also caused reduction in sDMA detected by the CS13222 antibody, and this reduction was similar in magnitude to that observed using the PRMT5 inhibitor EPZ15666 (Fig. 3*C*). A similar response to extracellular MTA was also observed in the MTAP-inducible cell line, MCM. MCM + Dox cells exposed to 100 μM extracellular MTA for 48 h lost crossreactivity to the CS13222 antibody (Fig. 3*D*), but internal MTA levels did not increase (Fig. 3*E*).

**Figure 4:**
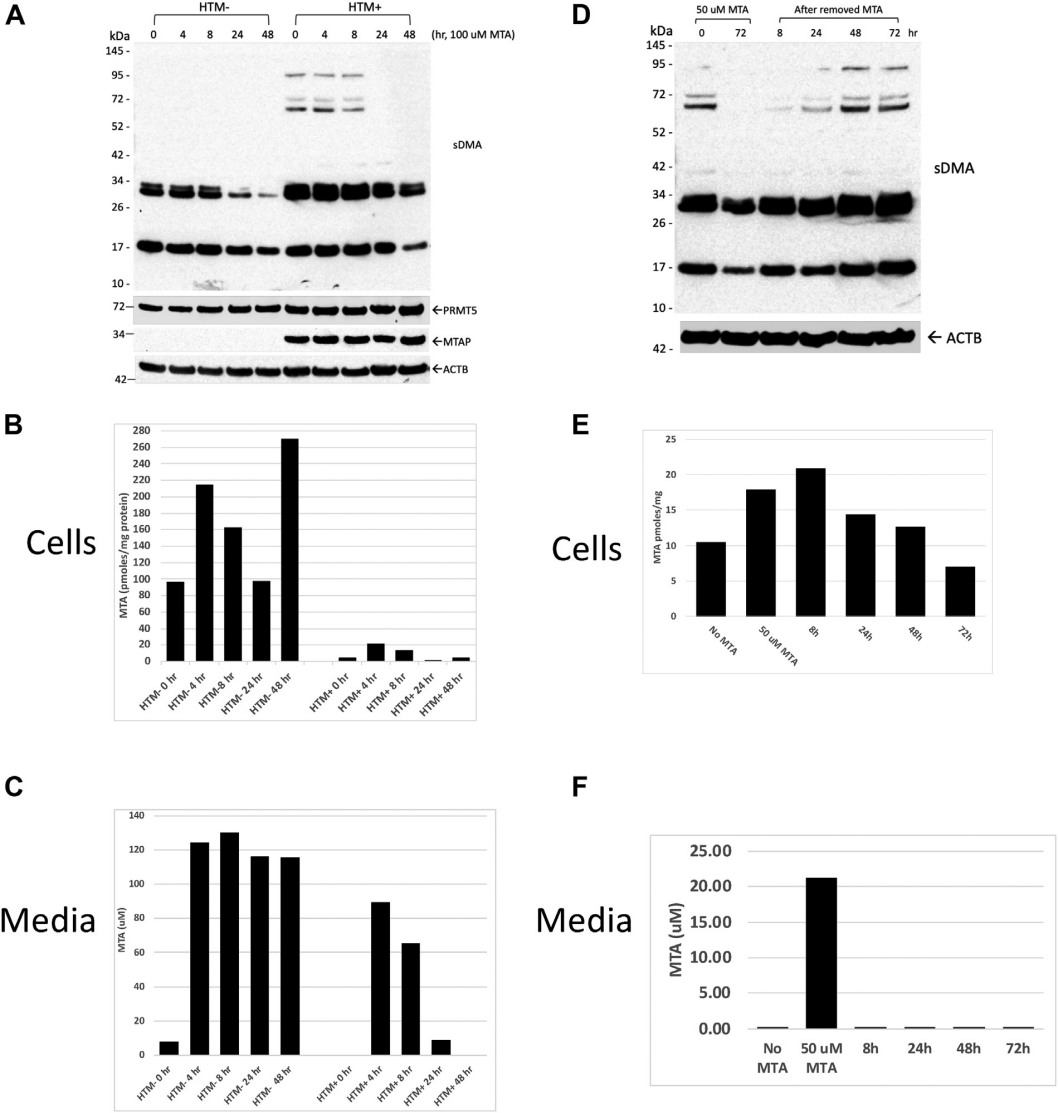
**Time course of sDMA inhibition and reactivation.***A*, Western blot showing effect of addition of 100 μM MTA over the course of 48 h. *B*, intracellular MTA as measured in same lysates. *C*, MTA in media at same time points. *D*, cells were exposed to 50 μM MTA for 72 h. Cells were then washed and replated in media lacking MTA for the period shown. Western blot shows sDMA levels. *E*, intracellular MTA as measured in same lysates as *D*. *F*, extracellular MTA measured at time of harvest. MTA, 5′-deoxy-5′-methylthioadenosine; sDMA, symmetric dimethylarginine.

We next examined the kinetics of MTA inhibition of sDMAylation. HTM− and HTM+ cells were incubated with media containing 100 μM MTA, and cells were harvested at various time points (Fig. 4*A*). In both cell types, protein sDMAylation was significantly decreased at 24 and 48 h. However, intracellular MTA in MTAP+ cells showed a small transient increase at four and eight time points but was back to near starting levels by 24 h (Fig. 4*B*). In the media, we found that the extracellular MTA rapidly decreased such that by 24 h, 87% of the MTA is gone, indicating that the MTAP+ cells are taking up MTA from the media and then breaking it down inside the cell (Fig. 4*C*). We did not observe any effect of MTA on the amount of PRMT5 in the cell (Fig. 4*A*). In another experiment, we examined the persistence of protein sDMAylation in HTM+ cells after MTAs were removed from the media (Fig. 4*D*). It took about 48 h for the protein sDMAylation to return to levels found in non–MTA-treated cells. In this experiment, we also measured MTA in cell lysates and media (Fig. 4, *E* and *F*). We observed about a twofold increase in intracellular MTA concentrations after exposure to extracellular MTA, which decreased back to baseline over time once the MTA was removed. However, it should be noted that the maximum amount of intracellular MTA observed in this experiment was only about 25% of that observed in HTM− cells.

Overall, our data show that there is a significant time lag between extracellular MTA addition or washout and alterations in intracellular protein sDMAylation. This does not seem to be consistent with a model in which MTA is a simple competitive inhibitor of PRMT5, especially given the long time required for remethylation when MTA is removed from the media. Another possibility that we entertained was that the MTA effect may be mediated by a signal transduction mechanism. Since MTA is structurally quite similar to adenosine (Fig. S7*A*), which is a key signaling molecule involved in a variety of physiological processes (20), we directly compared the effect of extracellular adenosine on sDMAylation in HTM+ cells (Fig. S7*B*). Unlike MTA, we did not observe any effect on protein sDMAylation at concentrations up to 320 μM. In addition, adenosine did not appear to interfere with response of sDMA to extracellular MTA. These findings suggest that MTA is not altering sDMAylation *via* signaling through adenosine receptors.

### MTAP+ cells can enhance sDMAylation in MTAP− cells in *trans*

**Figure 5:**
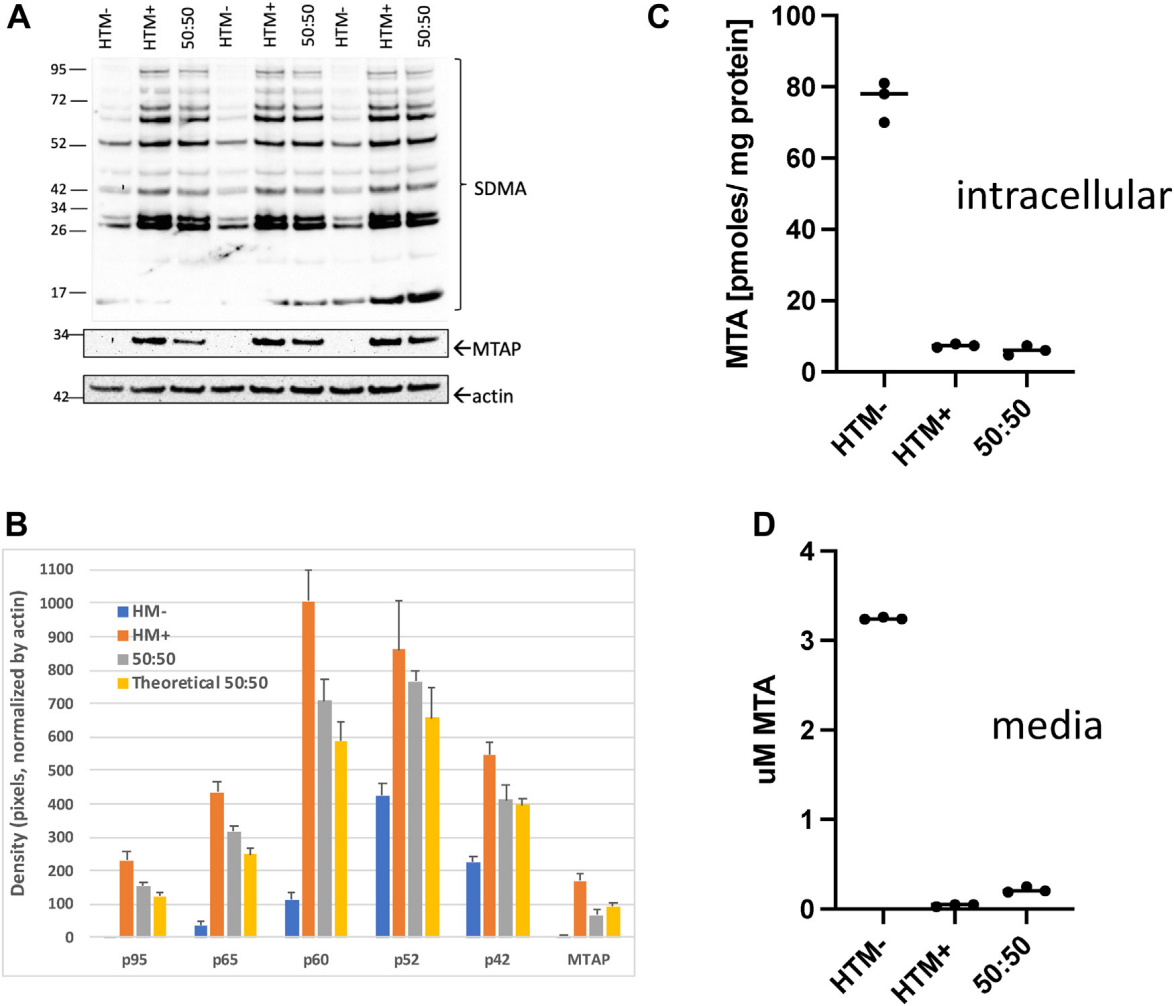
**Mixing experiment.***A*, Western blot showing protein sDMAylation in HTM−, HTM+, and a 50:50 mixture of both cells plated together. Cells were incubated for 48 h. Experiment was performed in triplicate. *B*, quantitation of bands shown with *arrows* in *A*. *C*, intracellular MTA in same lysates. *D*, MTA present in the media at time of harvest. MTA, 5′-deoxy-5′-methylthioadenosine; sDMA, symmetric dimethylarginine.

In tumors *in vivo*, MTAP− tumor cells are often in close proximity to MTAP+ stromal cells. Therefore, we compared protein sDMAylation in HTM+, HTM−, and cocultures of HTM+ and HTM− cells that were incubated together for 48 h (Fig. 5*A*). We observed that cocultures had sDMA levels more similar to that observed in HTM+ cells than HTM− cells. Specifically, quantitation of five differentially sDMAylated protein bands revealed that all five were elevated relative to the simple additive predictive model, with two being statistically significant (Fig. 5*B*). This is in sharp contrast to the MTAP protein, which was reduced slightly more than the expected 50%. Thus, the experiment indicates that HTM+ sDMA phenotype is dominant to the HTM− phenotype when the cells are grown together.

To explore this in more detail, we measured intracellular and extracellular MTA in the same samples (Fig. 5*C*). As observed previously, we found that HTM− cells had significantly higher levels of MTA in both the media and cell pellets compared with HTM+ cells. However, we observed no difference between HTM+ and the 50:50 sample with regard to intracellular MTA and only a very small increase in extracellular MTA in the 50:50 samples. The most likely explanation for these findings is that in the 50:50 mixture, the MTAP+ cells are taking up and metabolizing the MTA secreted from the MTAP− cells. These findings imply that the buildup of intracellular MTA in HTM− cells does not occur if extracellular MTA accumulation is inhibited.

### Identification of differentially sDMAylated proteins

To identify specific proteins that were differentially sDMAylated in response to MTAP status, we utilized label-free proteomics. Trypsin-digested protein extracts from duplicate samples HTM+ and HTM− cells were subjected to immunoprecipitation (IP) using the CS13222 antibody coupled to agarose beads, and the bound material was then subjected to LC–MS/MS. A total of 11,325 peptides from 3069 proteins were identified in at least one of the samples (Excel File S1). Of these, 142 peptides (1.3% of total) from 79 proteins (2.5% of total) contained DMA. DMA-containing peptides in 15 proteins exhibited at least a twofold differential in expression with a *p* value <0.1 between HTM+ and HTM− cells. Twelve of these proteins had DMA-containing peptides that were more abundant in HTM+ cells (Table 1), whereas three of the proteins had decreased levels. When examined for function, all 12 of these proteins either had proven or suspected RNA-binding activities. In addition, three of the proteins (FUBP1, FUBP2, and FUBP3) are all part of the same family of single-stranded DNA-binding proteins that act as gene enhancers (21).

**Table 1:** Proteins containing DMA that are differentially expressed in HTM+ *versus* MTM cells

Protein name	Gene	MTAP+/MTAP− ratio	*t* Test
Paraspeckle component 1	*PSPC1*	>1000	0.0016
Eukaryotic translation initiation factor 4H	*EIF4H*	>1000	0.006
Far upstream element–binding protein 1	*FUBP1*	7	0.01
Heterogeneous nuclear ribonucleoprotein Q	*SYNCRIP*	>1000	0.012
Plasminogen activator inhibitor 1 RNA-binding protein	*SERBP1*	>1000	0.018
Polyadenylate-binding protein 2	*PABPN1*	>1000	0.02
Proline- and serine-rich protein 2	*PROSER2*	56	0.02
Far upstream element–binding protein 3	*FUBP3*	>1000	0.03
Ataxin-2	*ATXN2*	>1000	0.05
UPF0696 protein C11orf68	*C11orf68*	4.5	0.09
Constitutive coactivator of PPAR-gamma-like protein 1	*FAM120A*	3.7	0.01
Far upstream element–binding protein 2	*KHSRP*	9.5	0.1

**Figure 6:**
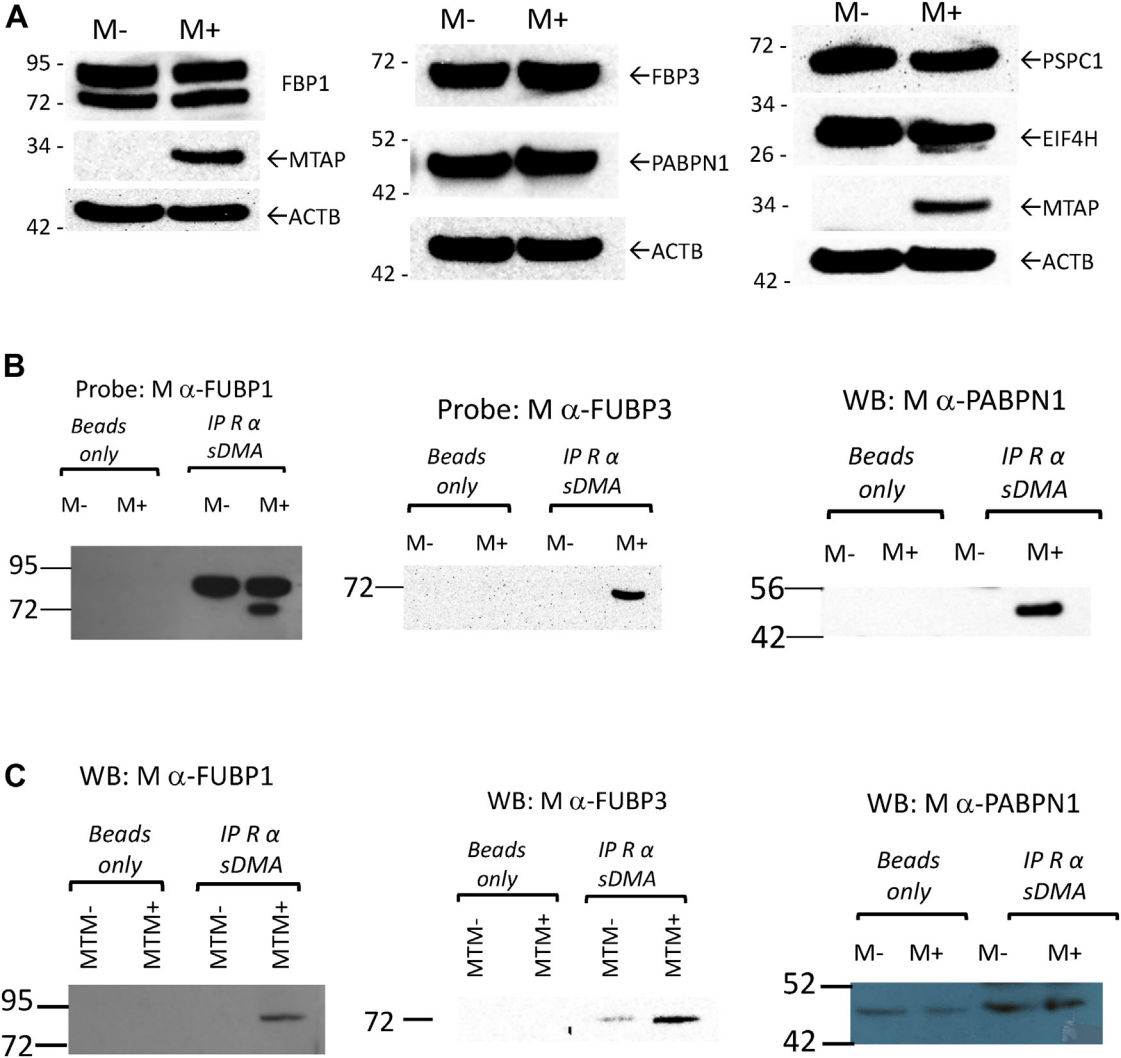
**Immunoprecipitation (IP) of sDMAylated proteins.***A*, Western blot showing levels of FUBP1, FUBP3, PABN1, PSPC1, and EIF4H in HTM+ (M+) and HTM− (M−) cell lysates. *B*, IP of indicated extracts using CS13222 antibodies to pull down sDMAylated proteins. Precipitated material was then subjected to Western analysis using indicated antibody. For each IP, a beads-only negative control is also shown. Note that for FUBP1, only lower band shows differential pulldown. *C*, same as *B*, but MTM cell extracts are used. sDMA, symmetric dimethylarginine.

To confirm that these proteins were differentially sDMAylated, monoclonal antibodies for five of the proteins including FUBP1, FUBP3, PSPC1, PABPN1, and EIF4H were obtained from commercial vendors and first used to examine protein levels in HTM+ and HTM− extracts. All the antibodies recognized proteins of the correct molecular weight, and we observed no differences in the overall amounts of these proteins in either MTAP+ and MTAP− cells (Fig. 6*A*). We examined the level of sDMAylation of the proteins by performing IP experiments. In these experiments, we used the CS13222 antibody coupled to beads to enrich for sDMAylated proteins, and then Western blots were probed using the various monoclonal antibodies to examine the relative amounts in HTM+ and HTM− cells. We were able to confirm differential sDMAylation in three of the proteins in the HTM+ and HTM− cell lysates (FUBP1, FUBP3, and PABPN1, Fig. 6*B*). In addition, we performed identical IPs using MCM− and MCM+ extracts and found that FUBP1 and FUBP3 were differentially sDMAylated in that cell type as well, although no enrichment of PABPN1 was seen (Fig. 6*C*).

### Extracellular MTA affects transcription from FUBP reporter plasmid

**Figure 7:**
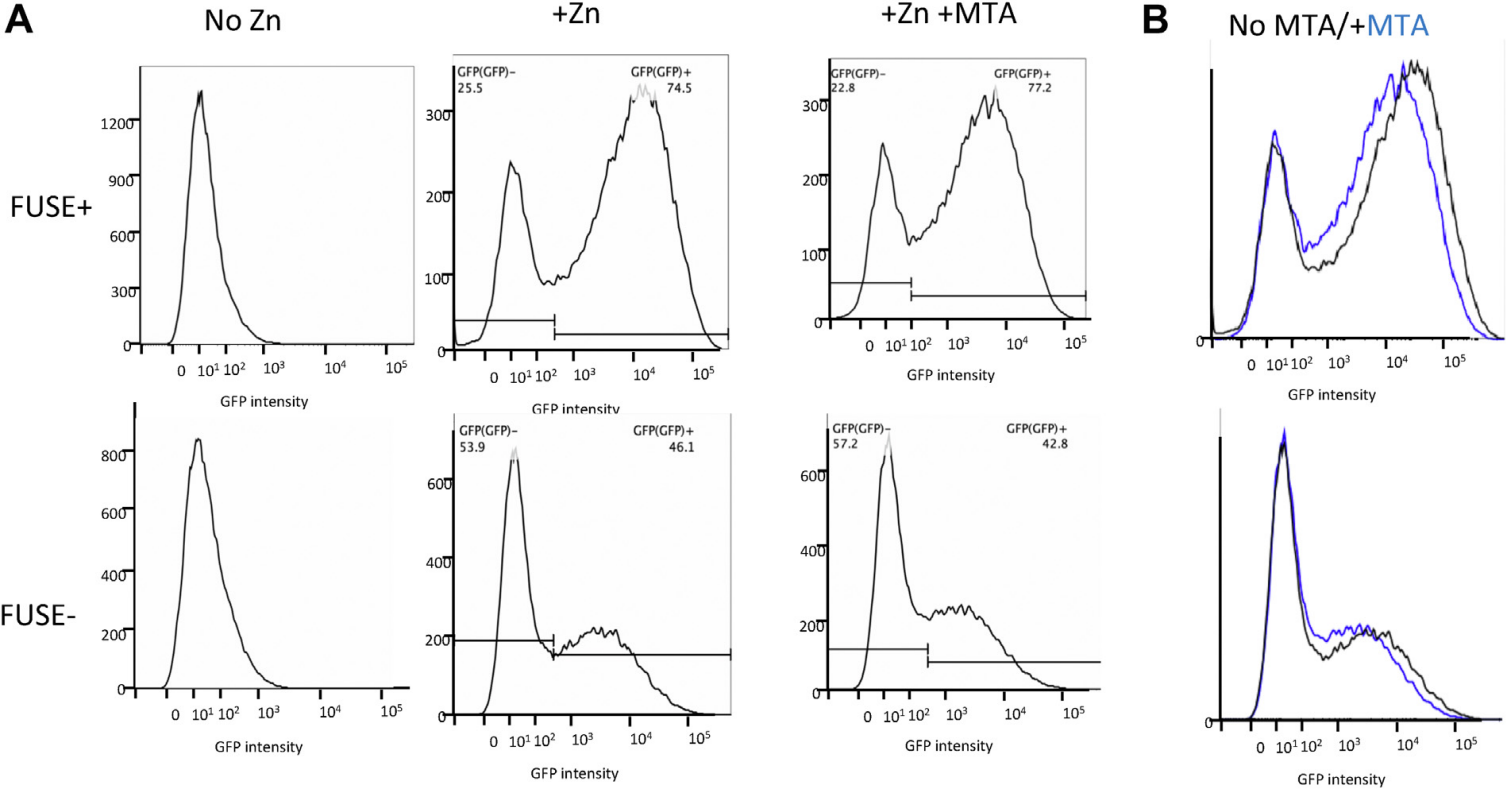
**FACS analysis of FUSE site function.***A*, cells transfected with reporter plasmid either containing or missing FUSE site were treated with zinc to induce the MT-I protomer driving a GFP promoter in either the presence or the absence of 100 μM MTA. *Horizontal bars* show windows used to assess parent induced *versus* uninduced cells. *B*, comparison of histograms with and without MTA treatment. FUSE, far upstream binding sequence element; MTA, 5′-deoxy-5′-methylthioadenosine.

FUBP proteins were initially identified as transcription factors that bind the far upstream binding sequence element (FUSE) DNA element located 1.4 kb upstream of the MYC promoter start site. It is thought that these proteins work by binding to single-stranded sequences that result from supercoiling caused by the torsional stress of RNA polymerase (22). To examine whether the differential sDMAylation of FUBP1 and/or FUBP3 had any functional effects, we utilized two reporter constructs previously created by the Levens’ laboratory to examine FUBP function (23). One construct (+FUSE) contains the FUSE site from human *MYC* gene placed between divergent metallothionein (*Mt-I*) promoters, whereas the other construct lacks the FUSE site (−FUSE). The promoter configuration induces negative supercoiling, causing the FUSE-site DNA to melt, allowing FUBP binding. The readout of the transcriptional strength of the promoter is GFP. For these studies, we chose to use Raji cells (MTAP+), as this cell line was known to express this construct robustly. Before doing the experiment, we confirmed that Raji cells had reduced sDMA when exposed to MTA (Fig. S8). We found that GFP expression was significantly more strongly induced by zinc at 5 h when the FUSE site was present (Fig. 7*A*), with 74.5% of the cells showing GFP induction, with a mean intensity of 8594 units, compared with only 46.1% induction with a mean of 3604 units in cells lacking the FUSE site. Importantly, upon treatment with 100 μM MTA for 48 h, we found that while the amount of induced cells did not change much (74.5% *versus* 77.2.), the mean intensity of the induced cells was 41% lower (7327 *versus* 4310 units) (Fig. 7*B*). These findings indicate that sDMAylation of FUBP is important for its gene activation function.

## Discussion

The experiments in this article explore the mechanism by which loss of *MTA*P causes reduced levels of protein sDMAylation and the identification of some specific proteins that are differentially methylated in response to MTAP. The relationship between MTAP loss and *PRMT5* was first identified *via* a series of three different shRNA screens, where knockdown of *PRMT5* resulted in a synthetic slow-growth phenotype when combined with loss of MTAP (12, 13, 14). Because our laboratory has a long history of studying MTAP, we first wanted to confirm these observations using various isogenic cell lines that our laboratory had developed. While we were able to confirm the reduction in sDMA caused by MTAP loss in three different pairs of isogenic MTAP+ and MTAP− cell lines, we also noted that many of the antibodies used to detect sDMAylated proteins in the earlier articles vary widely in terms of what proteins they recognize and their consistency from lot to lot. Many of the antibodies are created against the N-terminal tail of histone H4, but in our hands, we detected interactions with several different higher molecular weight bands that clearly were not H4. For this reason, we went on to validate the specificity of the CS13222 antiserum, which is a mixture of four different monoclonal antibodies that was the most consistent in terms of performance between lots. Using peptide competition studies, we showed that the bands recognized by this mixture could only be completed by a peptide that contains the sDMA modification but not containing other arginine modifications, confirming its specificity. In addition to antibodies, we also showed the reduction of sDMA in MTAP− HT1080 cells by directly measuring sDMA on total cell protein hydrolysates. These studies revealed two important facts: (1) the sDMA modification is rarer than the aDMA modification by a factor of 10 and (2) there is a 62% reduction in the sDMA levels in HTM− compared with HTM+ cells.

We were also able to confirm the synthetic slow-growth interaction between *PRMT5* knockdown and MTAP deletion in our HT1080 cells. However, we and Mavrakis *et al*. found that pharmacologic inhibition of PRMT5 affected both MTAP+ and MTAP− cells equally. This result is surprising, especially given that both the inhibitor and the shRNA seemed similarly effective in reducing sDMA (Fig. S4, *A* and *C*). One possible explanation is that PRMT5 protein may have some other cellular function in addition to its enzyme activity, and this interaction is somehow important to the genetic interaction with MTAP. We were unable to confirm that HT1080 MTAP− cells were more sensitive to either growth or sDMAylation in response to a pharmacologic inhibitor of AdoMet, despite showing that the inhibitor was highly effective in lowering AdoMet levels (Fig. S5). Although pharmacological inhibition of MAT2A has been reported to synergize with MTAP loss in some tumor cell lines, if one looks carefully at the data (see Fig. 2*B* in Ref. (24)), many MTAP-deleted cell lines do not show such synergy, suggesting that such interactions may be cell line specific.

A surprising finding described here was that addition of extracellular MTA to the media of MTAP*+* cells could cause decreased levels of sDMA, without a sustained increase in intracellular MTA. In a time course using HTM+ cells (Fig. 4), we did observe a slight transient increase in intracellular MTA levels, but this occurred at very early time points and was no longer present when sDMA started to decrease. A similar delay was also observed in the experiment in which MTA was removed from the media. Both lack of sustained MTA elevation and the delay between MTA addition/subtraction and change in sDMA was unexpected since MTA is a direct competitive inhibitor of AdoMet in by PRMT5-driven sDMAylation. In addition, the observation that a MAT2A inhibitor did not affect sDMAylation was also surprising. Given these findings, we entertained the alternative mechanistic hypothesis that MTA may be working by some sort of cell signaling mechanism, where delays are observed as the cells shift to a new epigenetic state. One obvious candidate for such a mechanism would be signaling through the adenosine receptors, as MTA has been shown to be an agonist of the A2_b_ receptor (25). However, we did not find any evidence that adenosine affected cellular sDMA levels either in the presence or in the absence of MTA, suggesting that if MTA is acting as a signaling molecule, it is independent of the known adenosine signal transduction system. It is also interesting to note that in prokaryotes, inhibition of the MTAP ortholog MTA nucleosidase, disrupts quorum sensing, an important signaling pathway involved in bacterial virulence (26). Further study will have to be done to understand the exact mechanism by which extracellular MTA can alter sDMAylation patterns in MTAP*+* cells.

Another important finding was the finding that the increase in sDMA observed in MTAP− cells could be almost entirely reversed by coculturing with MTAP*+* cells. This seems to happen because the MTAP*+* cells take up and metabolize the excess MTA excreted by MTAP− cells into the medium. We suspect that the excretion of MTA is largely a passive event, such that if extracellular MTA is low, the MTAP− cell can reduce its intracellular MTA by dilution. This finding is entirely consistent with the recent observation that in MTAP-deleted human glioblastoma tumor tissue, there is no increase in MTA (27), raising the idea that the amount of MTAP*+* stromal cells may be an important factor when trying to target MTAP loss therapeutically *via* PRMT5 inhibition. Smith *et al.* (28) have recently developed a small-molecule PRMT5 inhibitor that specifically targets the PRMT5–MTA complex. The idea behind this is that such an inhibitor would specifically target MTAP− tumors as their intracellular MTA is elevated. However, our findings and those of Barekatain *et al.* (27) suggest that *in vivo* there may be no elevation in intracellular MTA because of its being metabolized by MTAP+ stromal cells. Additional studies will need to be performed to see how universal is this phenomenon.

We used IP in combination with LC–MS/MS to look for proteins containing DMA that were differentially expressed in HTM+ and HTM− cells. Of the 12 proteins that were identified, three were members of FUSE family of single-stranded DNA-binding proteins. We confirmed that two of these proteins, FUBP1 and FUBP2, were differentially sDMAylated in MTAP+ *versus* MTAP− cells in both HT1080 and MCF-7 cells. FUBP proteins were initially identified because they bound to a transcriptional enhancer site upstream of the *Myc* promoter. However, they are unusual enhancer proteins in that they prefer to bind to single-stranded DNA. Mechanistically, they have been proposed to act as a sort of molecular “cruise control,” where they keep gene expression within a narrow window (29). Besides DNA, FUBP proteins also bind to RNA and are involved in processes such as splicing and control of translation (30). Dysregulation of FUBP1 expression is observed in a variety of cancers, although whether it is a tumor suppressor and oncogene are not entirely clear. In order to determine if sDMAylation of FUBP1 had any functional relevance, we made use of a FUSE:GFP reporter system developed by the Levins’ laboratory and used in Raji cells (21). We found that treatment of these cells with external MTA lowered sDMA levels and reduced the median level of induced GFP by 41%. This finding strongly suggests that the MTA-dependent sDMA modification found on FUBP1 and FUBP3 has functional significance.

In summary, our data confirm the relationship between MTAP loss and reduced protein sDMAylation in cells but suggest that the proposed mechanism of direct inhibition by MTA of PRMT5 is likely incorrect or at least oversimplified. In addition, we have identified FUBP1 and FUBP3 as proteins whose sDMAylation is sensitive to MTAP loss, and that decreased sDMAylation affects their efficiency as transcriptional modulators. Future efforts will need to be directed at understanding the importance of reduced protein sDMAylation on other proteins that are affected by loss of MTAP and how this plays a role in cancer development.

## Experimental procedures

### Cell lines and cell growth conditions extract preparation

Isogenic HTM+ and HTM− cell lines and isogenic MTAP*+/*MTAP− NIH 3T3 cells were created as previously described (6, 31). MCM cell line was created by cotransfecting in pTREtight:MTAP and a linear puromycin gene at a 20:1 ratio into MCF-7 Tet-On Advance cells (Clonetech) and selecting for puromycin-resistant clones. pTREtight:MTAP was made by inserting a BamH1/EcoRV fragment from pCR:sMTAP (7) into the pTRE-Tight vector (Clonetech). Induction of MTAP by Dox was determined by Western blot. All cells used in this study were grown in Dulbecco's modified Eagle's medium supplemented with 2 mM glutamine, 100 μg/ml streptomycin, 10% fetal bovine serum, and 250 μg/ml G418.

### Lysate preparation and Western blots

Whole cell lysates, SDS page, and Western blot analysis were performed as previously described (6). Nuclear and cytoplasmic fractions were isolated using the NE-PER kit (Thermo Fisher Scientific) according to the manufacturer’s instructions. The sDMA-recognizing antibodies are given in Fig. S2*A*. Other antibodies used were MTAP (catalog no.: SC-100782; Santa Cruz), actin (catalog no.: A4700; Sigma), PRMT5 (catalog no.: 2252; Cell Signaling Technology), FBP1 (catalog no.: sc-271241), FBP3 (catalog no.: sc-398466), eIF4H (catalog no.: sc-515265), PSPC1 (catalog no.: sc374181), and PABN1 (catalog no.: 66807-1-Ig; Proteintech).

### Peptide competition assay

One microgram per milliliter of sDMA antibody (catalog no.: 13222, Cell Signaling Technology) was added to PBS and had either no blocking peptide added or 5 mg/ml of various blocking peptides added. The unmodified sequence (derived from the N terminus of histone H4) was SGRGKGGKGC, and the arginine at position 3 was modified by either the monomethyl, asymmetric dimethyl, or symmetric dimethyl modification. Mixtures of antibody and peptide were incubated over night at 4 °C with agitation. Each preincubated antibody was diluted 1000× in 5% bovine serum albumin blocking buffer and added to the immunoblotting strips containing identical samples of HTM− and HTM+ cell lysates. Signals were visualized by chemiluminescent kit (Thermo Fisher Scientific) and Alpha Innotech image analyzer.

### Quantitative assessment of arginine methylation using protein hydrolysis and amino acid analysis

HTM−, HTM+, and HTM+ cells treated with 80 μM MTA for 3 days were harvested in total 10 ml of radioimmunoprecipitation assay (RIPA) buffer (Sigma) supplemented with protease inhibitor (Roche). Cell lysates were sonicated for 30 s on ice, and supernatant was collected by centrifugation at 13,000 rpm for 10 min at 4 °C. To hydrolyze protein, 10 mg of protein in RIPA buffer were added by same volume of 12 N HCl and heated at 110 °C for 24 h. The material was then dried under a gentle stream of nitrogen gas and dissolved with 200 μl of lithium loading buffer (Biochrom). The samples were reduced and extracted with sulfosalicylic acid as described previously (32). Peaks were measured using Biochrom 30 amino acid analyzer and quantitated by comparing to known external standards: NG-methyl-l-arginine acetate salt (catalog no.: M7033; Sigma), NG, NG-DMA dihydrochloride (catalog no.: D4268; Sigma), and NG, NG′-DMA di(p-hydroxyazobenzene-p’-sulfonate) salt (catalog no.: D0390; Sigma).

### PRMT5 shRNA studies

PRMT5 knockdown with shRNA was performed using a lentiviral vector (mission lentiviral transduction; Sigma). In brief, 5 × 10^4^ HTM− and HTM+ cells were seeded on 24-well plate. The following day, cells were replaced with 8 μg/ml Polybrene (Sigma)-containing media and lentiviral particles at eight multiplicity of infection of either pLKO.1-puro nontarget shRNA control transduction particle (catalog no.: SHC016V, CCGGGCGCGATAGCGCTAATAATTTCTC-GAGAAATTATTAGCGCTATCGCGCTTTTT) or mixture of two PRMT5-target shRNA transduction particles (catalog no.: TRCN0000303446; CCGGGGCTCAAGCCACCAATCTATGCTCGAGCATAGATTGGTGGCTTGAGCCTTTTTG and TRCN0000303447; CCGGCCCATCCTCTTCCCTATTAAGCTCGAGCTTAATAGGGAAGAGGATGGGTTTTTG) was added. Transduced cells were selected in media containing 1 μg/ml of puromycin and evaluated *Prmt5* knockdown by Western blot analysis using PRMT5 antibody (catalog no.: 2252; Cell Signaling Technology). For colony formation assays, shPRMT5-transfected HTM− and HTM+ cells were plated in triplicate on 24-well plates (2400 cells/well) and grown for 10 days. Colonies were stained with 0.5% crystal violet solution in 25% methanol and quantitated by Alpha Innotech image analyzer.

### Drug studies

EPZ01566 and PF9366 were obtained as dry powders (Medchemexpress) and diluted in dimethylsulfoxide at 10 and 200 mM, respectively, for stock solutions. Cell growth was measured by MTT (3-(4,5-dimethylthiazol-2-yl)-2,5-diphenyltetrazolium bromide) assay.

### AdoMet and MTA quantitation

AdoMet quantitative measurements were performed by LC–MS/MS as described previously (33). For MTA measurements, cell lysates (60 μg protein) or medium (100 μl) were spiked with stable isotope internal standard (^2^H_3_-MTA; Toronto Research Chemicals) and precipitated to remove protein by perchloric acid. After keeping the lysate at 4 °C for 30 min, the samples were centrifuged at 13,000 rpm for 15 min at 4 °C. The supernatant was collected neutralized to pH 7.2 by sodium phosphate buffer of pH 11.5 and passed through solid-phase extraction cartridges (Oasis HLB 1 cc 10 mg; Waters). Cartridges were then washed with water, and elution was done in 90% methanol containing 0.1% formic acid. For the analysis of cell lysates, the eluent was evaporated by using speed vacuum, reconstituted in 0.1% formic acid in water to concentrate it 10 times, and 10 μl was injected onto the LC column. For the analysis of media, the eluent was diluted six times in 0.1% formic acid, and 10 μl was injected onto the LC column.

For the chromatographic separation of metabolites, Waters Acquity UPLC H-Class was used with reverse-phase column (Waters Acquity BEH C18, 2.1 × 50 mm, particle size of 1.7 mm) with a flow rate of 0.6 ml/min under isocratic conditions at the temperature of 60 °C. Solvent A was 0.2% formic acid in water, and solvent B was 0.2% formic acid in acetonitrile (optima grade from Fisher Scientific). Mass spectrometric detection was done by triple quadrupole TSQ Quantum Access instrument from Thermo Scientific/Xcalibur software (version 2.1). The transitions used for each metabolite along with their collision energy (CE) are as follows: AdoMet (399.2 → 250.23, CE = 16), ^2^H_3_-AdoMet (402.2 → 250.23, CE = 16), MTA (Q: 298.05 → 136.04, CE = 20; q: 298.05 → 119.11, CE = 48), and ^2^H_3_-MTA (Q: 301.06 → 136.05, CE = 16; q: 301.06 → 119.11, CE = 48). For MTA, both quantifier (Q) and qualifier (q) transitions were recorded. Instrument parameters were as follows: electrospray ionization mode positive; spray voltage = 4000 V; capillary temperature = 380 °C; sheath gas pressure = 45; and auxillary gas pressure = 20.

### Immunoaffinity purification

HTM− and HTM+ cells were lysed in 20 mM Hepes, pH 8.0, 8 M urea, 1 mM activated orthovanadate, 2.5 mM sodium pyrophosphate, and 1 mM β-glycerophosphate, and lysates were sonicated and cleared by centrifugation. Proteins were alkylated and quenched with 5 mM dithiothreitol and 25 mM iodoacetamide, respectively. Lysates were digested with trypsin (Worthington) overnight and quenched with TFA to pH 2. Peptides were purified using reversed-phase Sep-Pak C18 cartridges (Waters) and eluted with 30% acetonitrile and 0.1% TFA. Eluents were frozen down at −80 °C overnight and lyophilized for 2 days in a standard lyophilization apparatus. Dried peptides were dissolved in IP buffer (50 mM Mops, 10 mM Na_2_HPO_4_, 50 mM NaCl, pH 7.2; Cell Signaling Technology). About 10 mg of peptides were then incubated with CS13222 antibody beads for 2 h at 4 °C on a rotator. Modified peptides were eluted from beads with 0.15% TFA and desalted on STAGE tips with C18 cores (Thermo Pierce). Enriched peptides were resuspended in 50 mM ammonium bicarbonate (Sigma) and subjected to a second digestion with trypsin (Promega) for 2 h, acidified with TFA, and desalted on STAGE tips. Purified peptides were dried under vacuum prior to LC–MS analysis.

### LC–MS/MS analysis for dimethylated peptides

LC–MS/MS was performed using a Thermo Ultimate 3000 RSLCNano UPLC system feeding into a Thermo Q Exactive Orbitrap mass spectrometer. LC was performed using a Thermo Scientific Easy-Spray C-18 PepMap 75 μm × 50 cm C-18 2 μm column in which a 142 min gradient of 2 to 20% (132 min) and 90% (10 min) acetonitrile with 0.1% formic acid was run at 300 nl/min at 50 °C. Effluent from the HPLC was then directly electrosprayed into the mass spectrometer. The parameters for the MS/MS analysis was essentially identical to that described (34). MS/MS fragmentation spectra were searched with MaxQuant software (35) (version 1.6.0.1) using Andromeda 1.5.6.0 against the Swiss-Prot human protein database (downloaded on April 24, 2019; 20,402 entries). The maximum missed cleavage rate was set to 5, which is important because methylation of arginine will make fragments resistant to trypsin cleavage. Dynamic modification was set to include monomethylation of arginine (+14.015 Da), demethylation of arginine (+28.03 Da), oxidation of methionine (+15.995), and acetylation of protein N terminus (+42.011 Da). Fixed modification was set to carbamidomethylation on cysteine residues (57.02 Da). The maximum parental mass error was set to 10 ppm, and the MS/MS mass tolerance was set to 0.02 Da. Peptides between 6 and 50 amino acids were analyzed. These values were log_2_ transformed, normalized to the corresponding median sample values, and significance will be determined using a premutation-based false discovery rate approach in the Perseus software package (36).

### IP Western blot

Cells were lysed in RIPA buffer containing 1× proteinase inhibitor cocktail, and lysate protein concentration was determined by Pierce’s bicinchoninic acid Protein Assay kit. One hundred microgram of lysate proteins were incubated with 2 μg of anti-sDMA antibodies (Cell Signaling Technology) on rocker platform overnight at 4 °C. About 50 microliter of protein A/G magnetic beads (Biomake.com) were added to antigen–antibody immunocomplex and incubated with rotation for 90 min at 4 °C. Magnetic bead pellets were washed five times with 1× TTBS (20 mM Tris, 150 mM NaCl, 0.1% Tween 20) containing 1× proteinase inhibitor cocktail on rotation for 5 min each at 4 C. After final wash, magnetic bead pellets were resuspended with 50 μl of 2× SDS sample buffer and heated at 95 to 100 °C for 5 min for Western blotting analysis.

### Transfection and fluorescence-activated cell sorting analysis

Raji cell line was purchased from American Type Culture Collection and cultured in RPMI1640 medium supplemented with 10% fetal bovine serum, 2 mM glutamine, 100 μg/ml penicillin, and 100 μg/ml streptomycin at 37 °C with 5% CO_2_ incubator. FUSE plasmids, pMT2-FUSE or pMT2 + FUSE, were obtained from Dr David Levens (21). Raji cells were transfected by electroporation with Amaxa Nucleofector II and Nucleofector Kit V reagents (Lonza Bioscience) following the manufacturer’s instruction and selected with 100 μg/ml hygromycin. To induce transcription, the transfected Raji cells were incubated in aforementioned RPMI medium with 90 μM ZnSO_4_. GFP fluorescence was measured with LSR II Flow Cytometer (BD Biosciences) and analyzed by using FlowJo software (FlowJo, LLC).

## Data availability

The data generated in this study are available upon request from the corresponding author.

## Supporting information

This article contains supporting information.

## Conflict of interest

The authors declare that they have no conflicts of interest with the contents of this article.
