# Supplementary material for: Extracellular 5′-methylthioadenosine inhibits intracellular symmetric dimethylarginine protein methylation of FUSE-binding proteins
Source: J Biol Chem. 2022 Aug 11;298(9):102367. doi: 10.1016/j.jbc.2022.102367 (PMC9467882; doi:10.1016/j.jbc.2022.102367)
Supplement: Supplemental Data [file mmc2.pdf]

A.

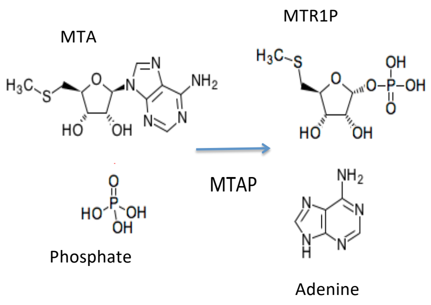
MTA
MTR1P
MTAP
Phosphate
Adenine

C.

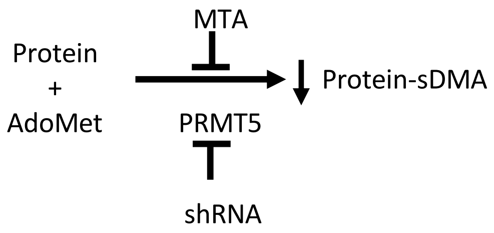
Protein
+
AdoMet
MTA
PRMT5
shRNA
Protein-sDMA

B.

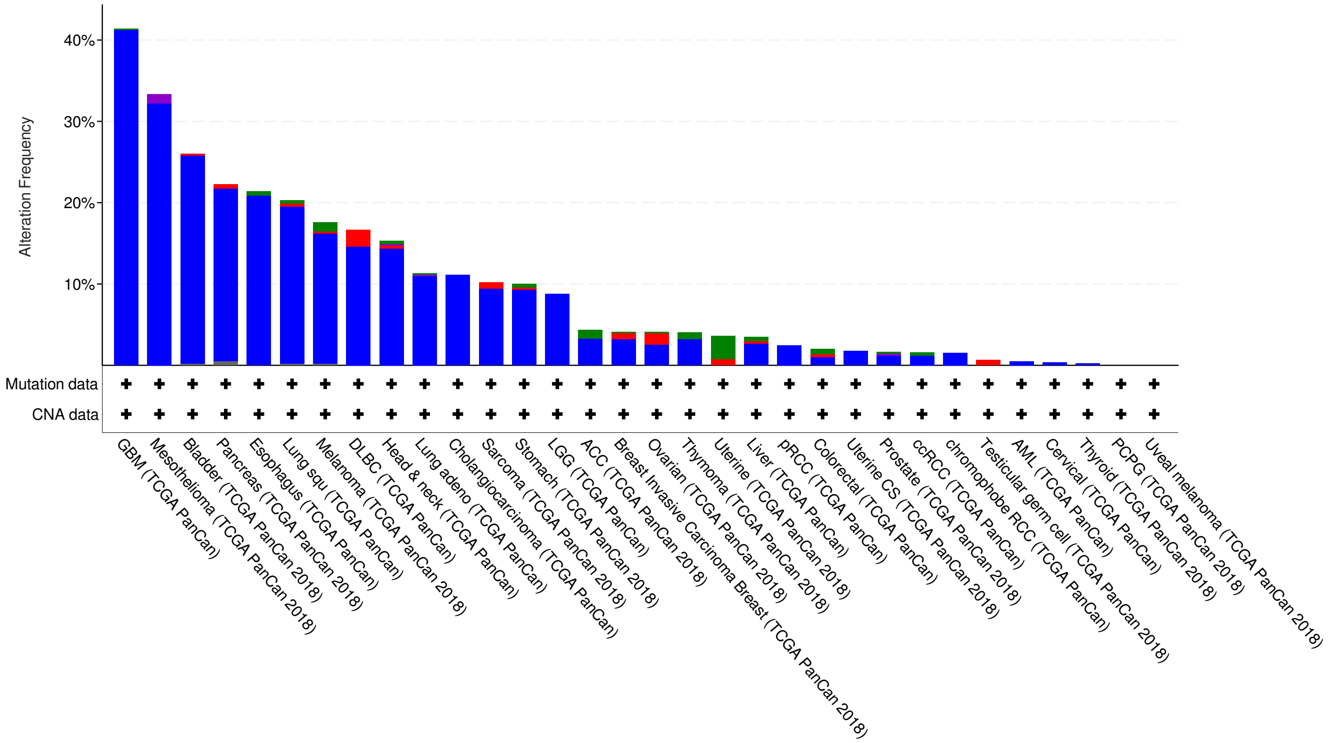
Alteration Frequency
40%
30%
20%
10%
Mutation data
CNA data
GBM (TCGA PanCan)
Mesothelioma (TCGA PanCan 2018)
Bladder (TCGA PanCan 2018)
Pancreas (TCGA PanCan 2018)
Esophagus (TCGA PanCan)
Lung squ (TCGA PanCan)
Melanoma (TCGA PanCan 2018)
DLBC (TCGA PanCan)
Head & neck (TCGA PanCan)
Lung adeno (TCGA PanCan)
Cholangiocarcinoma (TCGA PanCan)
Sarcoma (TCGA PanCan 2018)
Stomach (TCGA PanCan 2018)
LGG (TCGA PanCan)
ACC (TCGA PanCan 2018)
Breast Invasive Carcinoma Breast (TCGA PanCan 2018)
Ovarian (TCGA PanCan 2018)
Thymoma (TCGA PanCan 2018)
Uterine (TCGA PanCan 2018)
Liver (TCGA PanCan)
pRCC (TCGA PanCan)
Colorectal (TCGA PanCan)
Uterine CS (TCGA PanCan 2018)
Prostate (TCGA PanCan 2018)
ccRCC (TCGA PanCan)
chromophobe RCC (TCGA PanCan)
Testicular germ cell (TCGA PanCan 2018)
AML (TCGA PanCan)
Cervical (TCGA PanCan)
Thyroid (TCGA PanCan 2018)
PCPG (TCGA PanCan 2018)
Uveal melanoma (TCGA PanCan 2018)

Sup. Fig 1

A.

Antibodies	antigen	Identifier	Source figs
Polyclonal Rabbit Anti-Symmetric Dimethyl Arginine (SDMA) (SYM10)	peptide containing four symmetrical dimethyl-arginine-glycine repeats, Recognizes proteins that contain multiple symmetrically dimethyl arginines	Millipore Cat# 07-412	6 bands including p80-Coilin and the Sm protein B, B' and D.
Polyclonal Rb Anti-H4R3 Symmetric Dimethyl (H4R3me2s)	Peptide 1-10 aa of histone H4 (conjugated to KLH) in which Arg3 is di-methylated	Epigentek Cat# A-3718	1 band (Hela Cell) 14Kd, Dot blot showing specificity to modified peptide.
Polyclonal Rb Anti-H4R3 Symmetric Dimethyl (H4R3me2s)	Peptide 1-10 aa of histone H4 (conjugated to KLH) in which Arg3 is di-methylated	Dr. Allis #8039 Lot# 8060355	multiple bonds
Polyclonal Rb Anti-H4R3 Symmetric Dimethyl (H4R3me2s)	Peptide of Histone H4 aa 1-100 (symmetric di methyl R3) conjugated to Keyhole Limpet Haemocyanin (KLH).	Abcam Cat# ab5823	1 band (14 kD) H4R3me2s
Monoclonal Mix Rb Symmetic Dimethyl Arginine	Unknown antigen, but possibly H4 aa1-10.	Cell Signaling Cat#13222	Multiple Bands.
Monoclonal Mouse anti-H4R3 Symmetric Dimethyl (H4R3me2s)	Unknown. Product sheet claims to recognize R3me2s in H2A and H3, as well as "uncharacterized bands".	Millipore Cat #MABE364	1 band (14 kD)
Rb anti-Mono-Methyl Arginine (MMe-R) (D5A12)	mono-methyl arginine, prefers Arg-Gly-Gly motifs, does not cross-react with di-me arginine or unmethylated arginine.	Cell Signaling Cat #8711	multiple bonds
Rb anti-Asymmetric Di-Methyl Arginine Motif (ADMA)	React proteins with Asymmetrically Di-Me Arginine residues, not cross-react with monomethyl, symmetric methyl arginine, or methyl lysine residues.	Cell Signaling Cat #13522	multiple bonds

B.

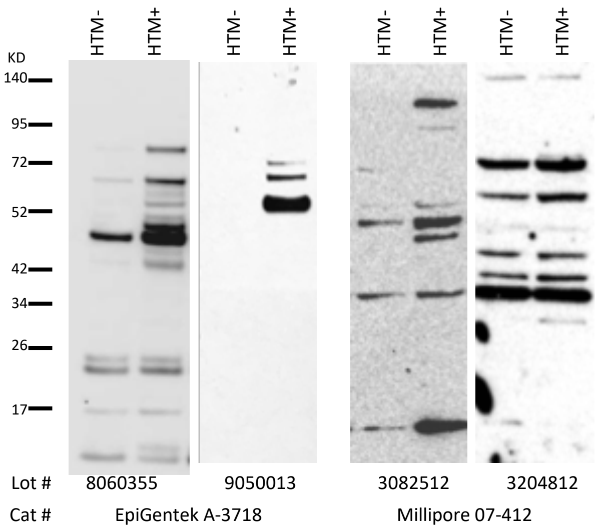

Sup. Fig 2

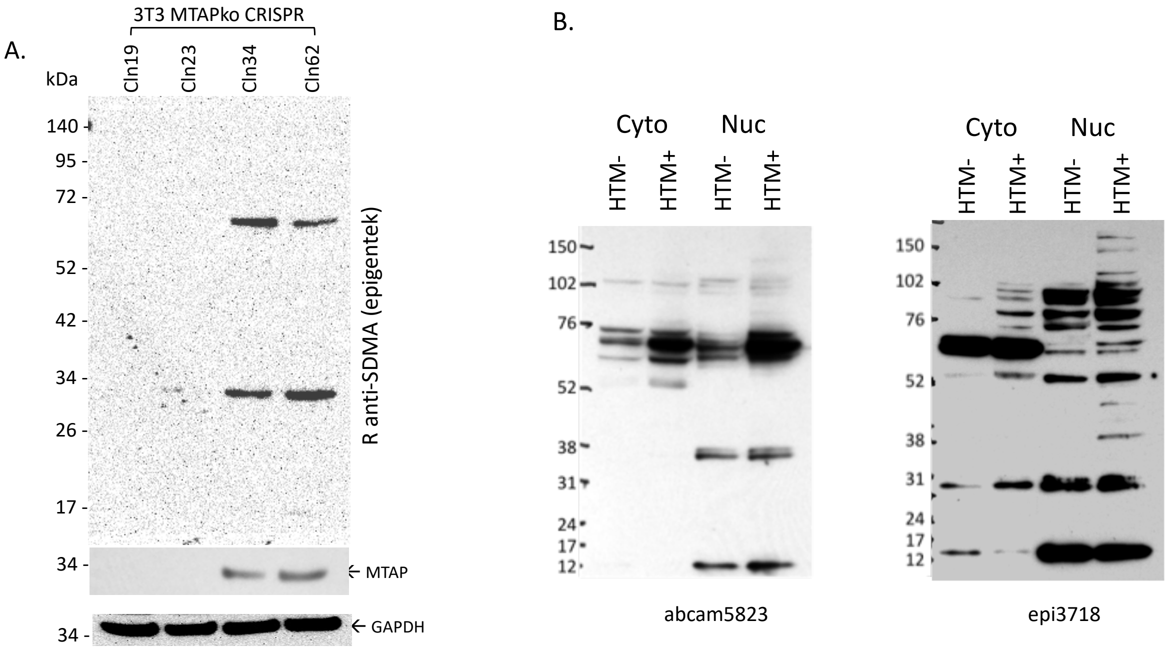
A.
3T3 MTAPko CRISPR
Cln19
Cln23
Cln34
Cln62
kDa
140
95
72
52
42
34
26
17
R anti-SDMA (epigentek)
34
MTAP
34
GAPDH
B.
Cyto
Nuc
HTM-
HTM+
HTM-
HTM+
150
102
76
52
38
31
24
17
12
abcam5823
Cyto
Nuc
HTM-
HTM+
HTM-
HTM+
150
102
76
52
38
31
24
17
12
epi3718

Sup Fig 3

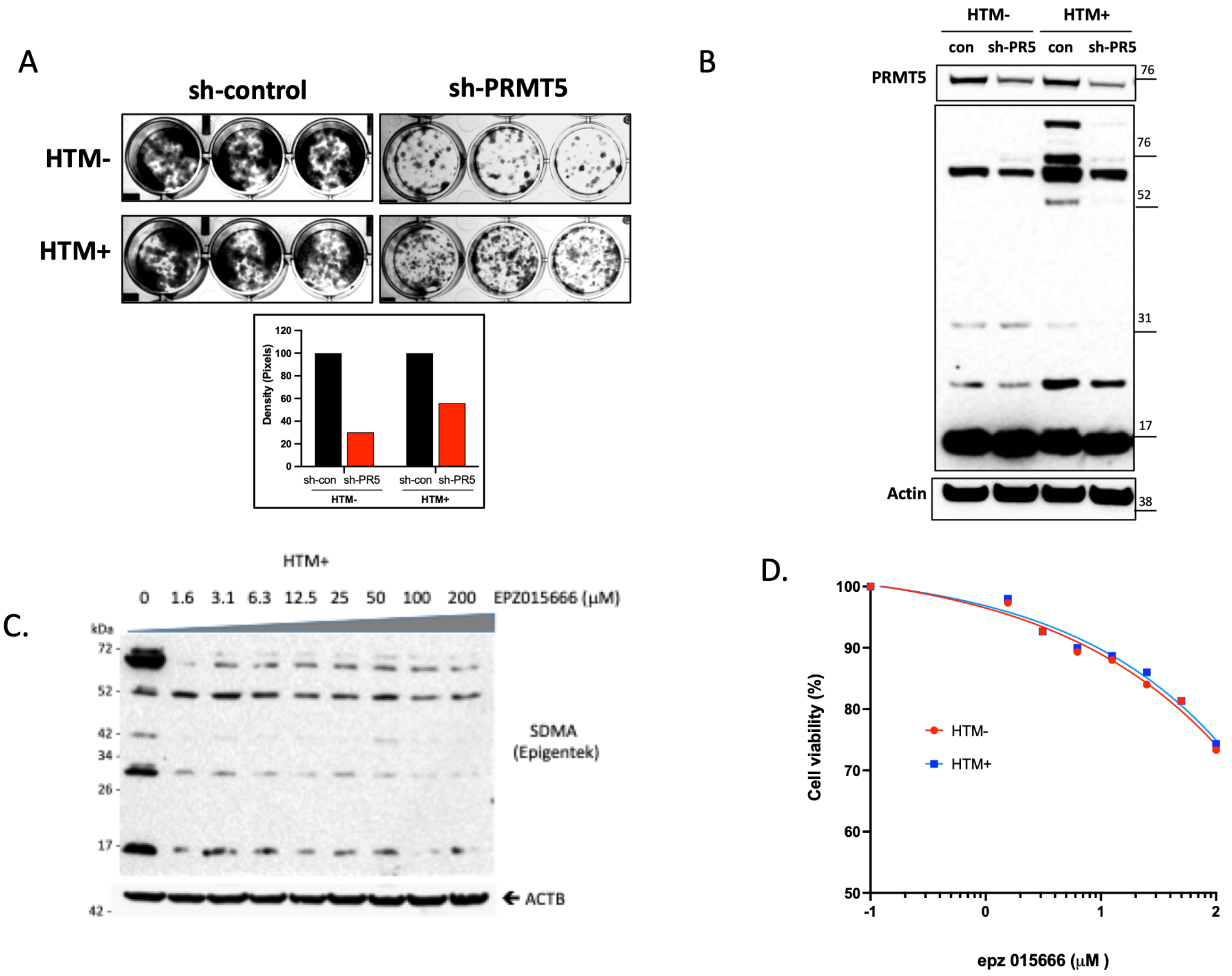
A
sh-control
sh-PRMT5
HTM-
HTM+
Density (Pixels)
120
100
80
60
40
20
0
sh-con
sh-PR5
sh-con
sh-PR5
HTM-
HTM+
B
HTM-
HTM+
con
sh-PR5
con
sh-PR5
PRMT5
76
76
52
31
17
Actin
38
C.
HTM+
0
1.6
3.1
6.3
12.5
25
50
100
200
EPZ015666 (μM)
kDa
72
52
42
34
26
17
42
SDMA
(Epigentek)
ACTB
D.
Cell viability (%)
100
90
80
70
60
50
-1
0
1
2
HTM-
HTM+
epz 015666 (μM)

Sup Fig 4

A.

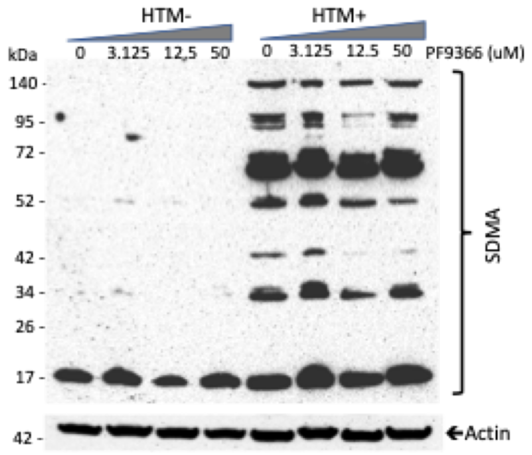
HTM-
HTM+
kDa
0 3.125 12,5 50 0 3.125 12.5 50 PF9366 (uM)
140 -
95 -
72 -
52 -
42 -
34 -
26 -
17 -
SDMA
42 -
Actin

B.

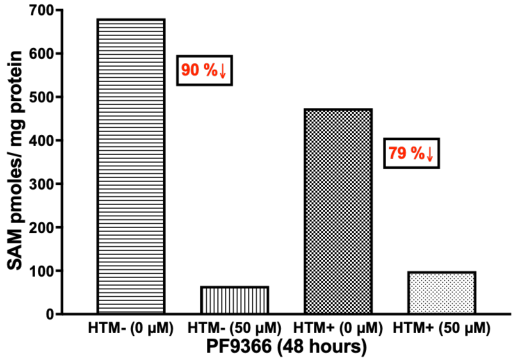
SAM pmoles/ mg protein
700
600
500
400
300
200
100
0
90 %↓
79 %↓
HTM- (0 µM)
HTM- (50 µM)
HTM+ (0 µM)
HTM+ (50 µM)
PF9366 (48 hours)

C.

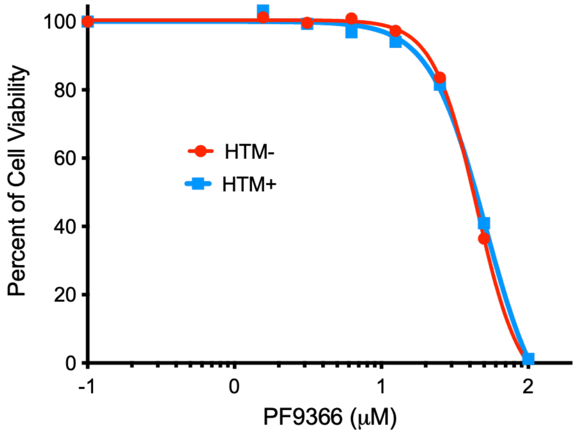
Percent of Cell Viability
100
80
60
40
20
0
HTM-
HTM+
-1
0
1
2
PF9366 (µM)

Sup Fig 5

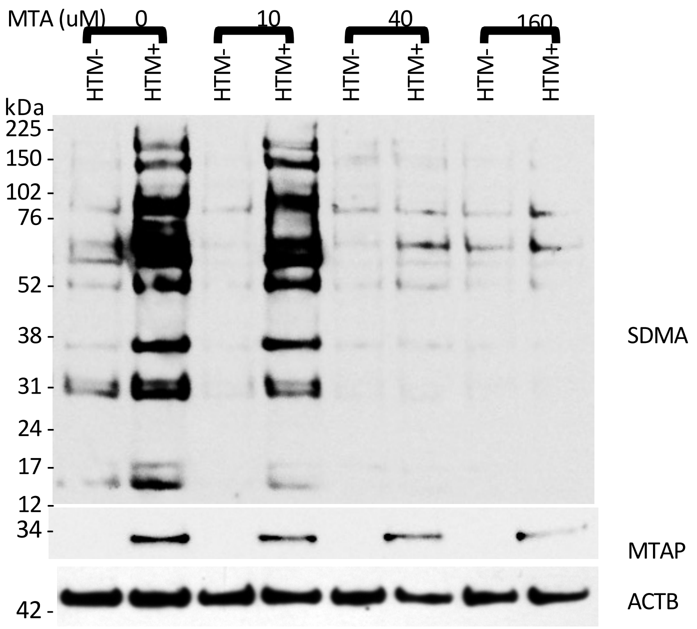
MTA (uM)
0
10
40
160
HTM-
HTM+
HTM-
HTM+
HTM-
HTM+
HTM-
HTM+
kDa
225
150
102
76
52
38
31
24
17
12
34
42
SDMA
MTAP
ACTB

Sup Fig 6

A.

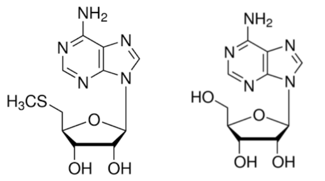

MTA Adenosine

B.

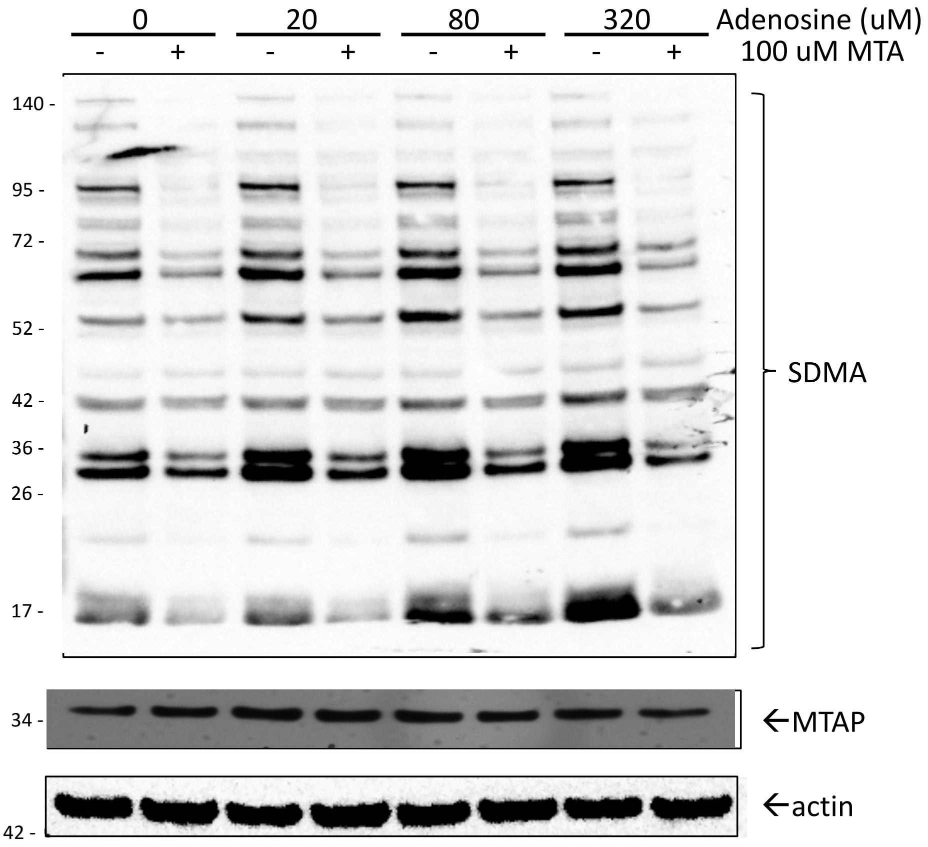

Sup.Fig 7

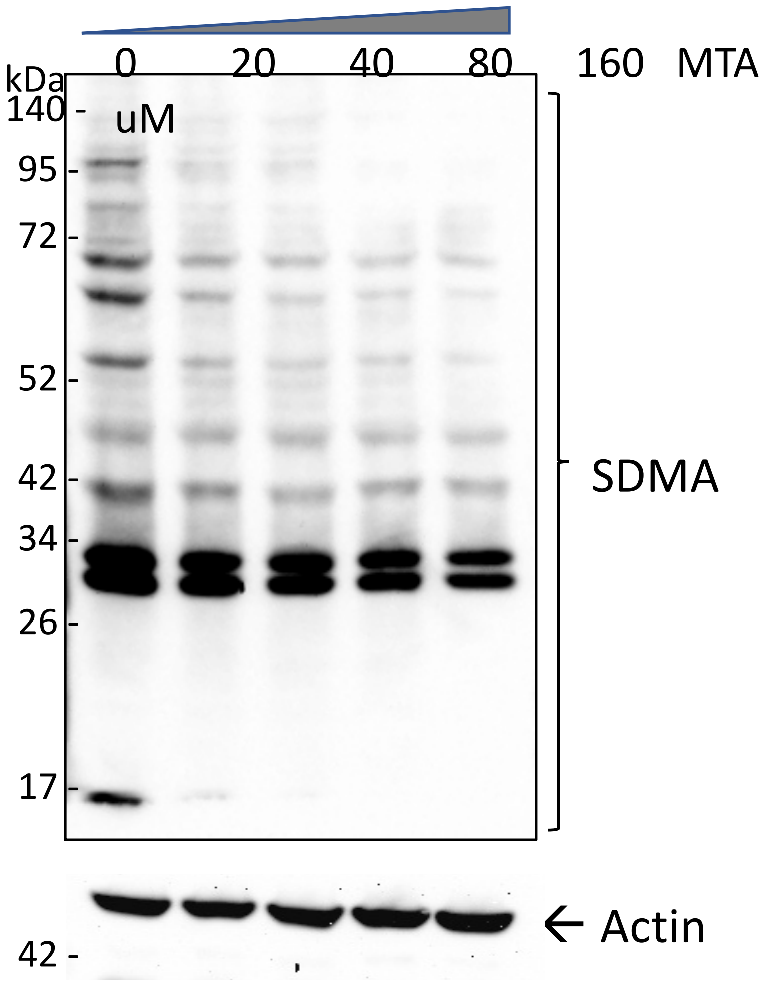

Sup Fig 8
